# Improved Statistical Methods Enable Greater Sensitivity in Rhythm Detection for Genome-Wide Data

**DOI:** 10.1371/journal.pcbi.1004094

**Published:** 2015-03-20

**Authors:** Alan L. Hutchison, Mark Maienschein-Cline, Andrew H. Chiang, S. M. Ali Tabei, Herman Gudjonson, Neil Bahroos, Ravi Allada, Aaron R. Dinner

**Affiliations:** 1 Medical Scientist Training Program, University of Chicago, Chicago, Illinois, United States of America; 2 Graduate Program in the Biophysical Sciences, University of Chicago, Chicago, Illinois, United States of America; 3 James Franck Institute, University of Chicago, Chicago, Illinois, United States of America; 4 Center for Research Informatics, University of Illinois at Chicago, Chicago, Illinois, United States of America; 5 Department of Neurobiology, Northwestern University, Evanston, Illinois, United States of America; 6 Department of Chemistry, University of Chicago, Chicago, Illinois, United States of America; University of California San Diego, UNITED STATES

## Abstract

Robust methods for identifying patterns of expression in genome-wide data are important for generating hypotheses regarding gene function. To this end, several analytic methods have been developed for detecting periodic patterns. We improve one such method, JTK_CYCLE, by explicitly calculating the null distribution such that it accounts for multiple hypothesis testing and by including non-sinusoidal reference waveforms. We term this method empirical JTK_CYCLE with asymmetry search, and we compare its performance to JTK_CYCLE with Bonferroni and Benjamini-Hochberg multiple hypothesis testing correction, as well as to five other methods: cyclohedron test, address reduction, stable persistence, ANOVA, and F24. We find that ANOVA, F24, and JTK_CYCLE consistently outperform the other three methods when data are limited and noisy; empirical JTK_CYCLE with asymmetry search gives the greatest sensitivity while controlling for the false discovery rate. Our analysis also provides insight into experimental design and we find that, for a fixed number of samples, better sensitivity and specificity are achieved with higher numbers of replicates than with higher sampling density. Application of the methods to detecting circadian rhythms in a metadataset of microarrays that quantify time-dependent gene expression in whole heads of Drosophila melanogaster reveals annotations that are enriched among genes with highly asymmetric waveforms. These include a wide range of oxidation reduction and metabolic genes, as well as genes with transcripts that have multiple splice forms.

## Author Summary

Much biomedical research focuses on how the expression of genes changes over time. Many genes’ activities vary periodically. For example, circadian rhythms repeat daily with the light-dark cycle. Understanding how such rhythms couple to biological processes requires statistical methods that can identify cycling time series in typical genome-wide data. In this paper, we improve on a method used to identify cycling time series by better estimating the statistical significance of periodic patterns and, in turn, by searching for a wider range of patterns than traditionally investigated. We apply these methods to a compilation of data on gene expression in fruit flies, an important model organism. We find that our method allows us to discover rhythmic biological activities that the other methods tested are unable to reveal.

## Introduction

Rhythmic behavior is ubiquitous across the spectrum of life [1–4]. Diverse fundamental biological functions such as cell division, energy metabolism, and sleep are periodic, and a growing body of evidence implicates temporal dysregulation as a contributing factor to depression, neurodegeneration, cardiovascular disease, and metabolic disorders in higher organisms [5–9]. Arguably the most well-studied periodic patterns are circadian rhythms: oscillatory changes in gene expression, metabolism, physiology, and behavior with approximately 24-hour (24 h) periods that enable organisms to anticipate and respond to daily changes in their environment, such as nutrient accessibility, temperature, and light [10–13].

Circadian rhythms arise from innate clocks. The components of the core clock are well characterized and are strongly conserved across a wide range of species [14, 15]. However, it remains to be determined how this clock couples to other molecular processes. Moreover, these interactions are likely to depend on tissue type and environmental conditions [2, 7, 11, 16, 17]. There is thus a need to identify molecular profiles that cycle and to characterize them as a function of conditions. The advent of high throughput methods for measuring gene expression now makes transcriptome-wide studies of this nature possible. Previous work suggests that hundreds, possibly thousands, of genes are regulated by circadian clocks [10, 15, 18].

Despite the decreasing cost of measuring transcript levels, profiling time series genome-wide continues to present formidable challenges: tissue-specific samples are difficult to collect, and, in contrast to imaging, measuring transcript levels is destructive in nature, requiring separate samples for each time point. As a result, gene expression time series are typically sparsely sampled (e.g., every 2–4 hours (h) in circadian studies), often without multiple measurements per time point, which we refer to here as “replicates”. These experimental limitations result in low signal-to-noise ratios that prevent straightforward identification of cycling gene expression.

Quantitative methods are thus needed to identify rhythmic time series from minimal data with statistical confidence. These methods can aid researchers in assessing the tradeoffs between the amount of data acquired, statistical precision, and breadth of biological discovery. While a number of different methods have been proposed for identifying cycling time series [19–30], further analysis is needed to guide selection of the best method(s) for a given situation and to aid in design of improved computational methods and further experiments.

In this paper, we improve on the JTK_CYCLE method [26]. The original method uses a conservative estimation for its *p*-values and a cosine as its only reference waveform. Here, we introduce a procedure, empirical JTK_CYCLE with asymmetry search, that provides accurate empirically-calculated *p*-values for arbitrary waveforms. We test its performance for detecting rhythms in simulated data and a circadian metadataset [27] against other algorithms: cyclohedron test [20, 21], address reduction [22, 23], stable persistence [24, 25], F24 [31, 32], and one-way analysis of variance (ANOVA) [27]. The simulated data allow us to examine how performance varies with sampling density, number of replicates and/or periods, noise level, and waveform. Most methods provide accurate rhythm detection when sampling density is high and noise is low. However, we find that the choice of method significantly affects rhythm detection when data are limited and/or noisy. In particular, JTK_CYCLE, F24, and ANOVA consistently outperform the other methods and offer distinct advantages for certain types of data. Our improved method, empirical JTK_CYCLE with asymmetry search, performs best of all for data that include asymmetric waveforms. Application of our improved method, empirical JTK_CYCLE with asymmetry, to a metadataset of whole head *D. melanogaster* circadian microarrays [27] reveals a strong lights-on peak in expression for genes involved in glutathione metabolism, high enrichment for genes involved in oxidation reduction, many more metabolic genes cycling than previously appreciated, and rhythmic genes with transcripts that have alternative splicings.

## Methods

### Overview

The methods that we consider are general and can be applied to detecting periodic behavior in any context, but we describe them here in terms of searching for circadian rhythms in gene expression for clarity. The methods that we test are cyclohedron test [20, 21], address reduction [22, 23], stable persistence [24, 25], F24 [31, 32], one-way analysis of variance (ANOVA) [27], and JTK_CYCLE [26]. We describe each briefly below and note specific features; additional details can be found in the references introducing the methods.

The methods can be broadly categorized as tests with and without reference waveforms. Cyclohedron test, address reduction, stable persistence, and ANOVA seek to identify patterns without specifying the waveform *a priori*. Address reduction, cyclohedron test, and stable persistence test for monotonicity. ANOVA compares the means of different time points with their variances to determine if differences are significant.

In contrast, F24 and JTK_CYCLE compare the time series in question to a reference waveform, which is typically sinusoidal. These methods also test for a specific period. As mentioned above, here we assume a period of 24 h, but the period of the reference can be varied, in the same manner that the phase can be varied, to search for rhythms on other time scales.

#### Cyclohedron test

Cyclohedron test [20, 21] maps data to a cyclohedron and joins data points into sets according to their adjacency in rank-ordering. Monotonicity is quantified by how the sizes of the sets scale as more data points are included. Cyclohedron test has an exact null distribution computable from a generating function. The domain of test statistics increases very quickly with the number of data points, however, so Monte Carlo (MC) sampling, in which representations of the null model are randomly generated and evaluated, is required to estimate *p*-values if there are more than approximately 20 time points due to the computational cost of the generating function.

#### Address reduction

Address reduction [22, 23] measures the entropy of the dataset by comparing the rank-ordering of adjacent time points. Low entropy data are monotonic and score higher in the method. The null distribution for address reduction is estimated by MC sampling.

#### Stable persistence

In stable persistence [24, 25], local minima are paired with surrounding local maxima, and the “persistences” of these features are characterized by the differences in ranks of the paired extrema. A hierarchy of such features is established, and the test compares the global persistence to local ones. In this way, stable persistence tries to robustly assess overall monotonicity of a time series. The null distribution for stable persistence is estimated by MC sampling.

#### Analysis of variance (ANOVA)

One-way ANOVA is a standard statistical test of the equivalence of means in several groups. In this case, each time point is a different group, and ANOVA is equivalent to testing for any statistically significant variation across the time points. Because expression measurements are averages over many cells and different time points come from different samples (as the measurement is destructive), only synchronized, consistent variation across all samples can generate a statistically significant trend. By this reasoning, significant changes in expression across time points can be attributed to time-dependent expression within the population, such as circadian rhythms. ANOVA tests for these time-dependent changes in expression. ANOVA has an exact null distribution derived from an assumption of normally distributed data; unlike the other five methods, however, ANOVA requires replicates to estimate the variance of experimental measurements at each time point.

#### 24-hour Fourier projection (F24)

F24 [31, 32] assesses periodicity by focusing on the 24 h period of the Fourier transform of the data. The test statistic for F24 is the projection of the data onto the 24 h Fourier basis function, and the null distribution is obtained by recomputing the test statistic over repeated random permutations of the data. The phase is determined by projecting the data onto the cosine part of the Fourier basis function and finding the optimal phase for the projection. We find that the null distribution can be modeled by the Gamma distribution (S1 Fig.), which we parameterize from the mean and variance of the null distribution. We estimate the null distribution from a small number of permutations (usually 100). This allows more rapid and precise computation of *p*-values than can be obtained by standard permutation. Testing periods other than 24 h is accomplished simply by changing the period of the Fourier basis function used to compute the test statistic.

#### Jonckheere-Terpstra-Kendall cycle (JTK_CYCLE)

**Fig 1:**
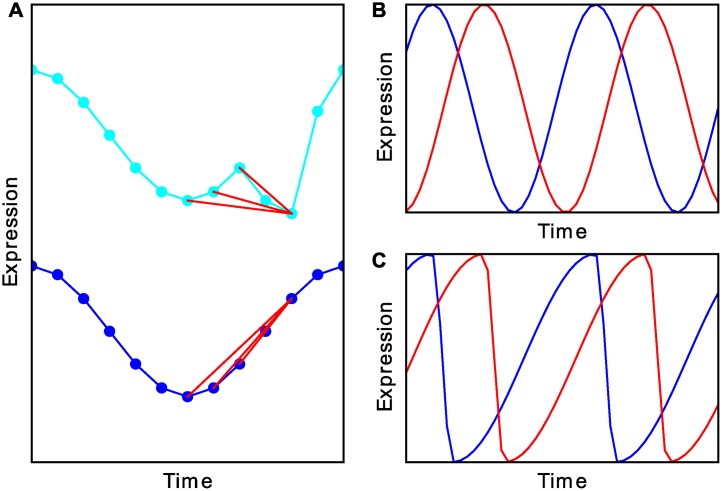
JTK_CYCLE compares all possible pair relations for a time series to those for a reference waveform. (A) JTK_CYCLE tests for pairwise agreement between a reference (blue) and a signal (cyan) time series. Three discordant pairwise relationships are indicated by red lines. (B) The previous implementation compared a time series to a set of phase-shifted cosines. (C) We add a set of asymmetric waveforms to the reference. An example is shown here with the same phases as in A.

JTK_CYCLE [26] computes the Kendall *τ* rank correlation coefficient between the data and a reference function over a range of possible reference function phases. For two time series $\vec{x}=(x_{1},x_{2},\ldots ,x_{n})$ and $\vec{y}=(y_{1},y_{2},\ldots ,y_{n})$,

$$\begin{array}{c} \tau \left(\vec{x},\vec{y}\right)=\frac{\sum_{1\leq i<j\leq n}\text{sgn}\left(x_{j}-x_{i}\right)\cdot \text{sgn}\left(y_{j}-y_{i}\right)}{\frac{1}{2}n\left(n-1\right)}\; \end{array}$$ (1)

where sgn(*x*) is −1 if *x* < 0 and +1 if *x*>0. The numerator is the number of pairs that vary concordantly between the two time series minus the number that vary discordantly (Fig. 1A). Every possible pair is included, not just ones between neighboring points in the time series. The denominator is the total number of pairs, which normalizes the value of *τ* to the range [−1, 1]. Perfectly correlated series score *τ* = 1, perfectly anti-correlated series score *τ* = −1, and uncorrelated series score *τ* = 0. Like the cyclohedron test, the null distribution for JTK_CYCLE can be computed exactly from a generating function [33], although again the test statistic domain grows quickly with time series size (becoming impractically large at 100–200 time points with present computing power). However, for large time series the JTK_CYCLE null distribution is approximately normal, allowing for a convenient, fast *p*-value estimate.

### Improvements to the JTK_CYCLE method

**Fig 2:**
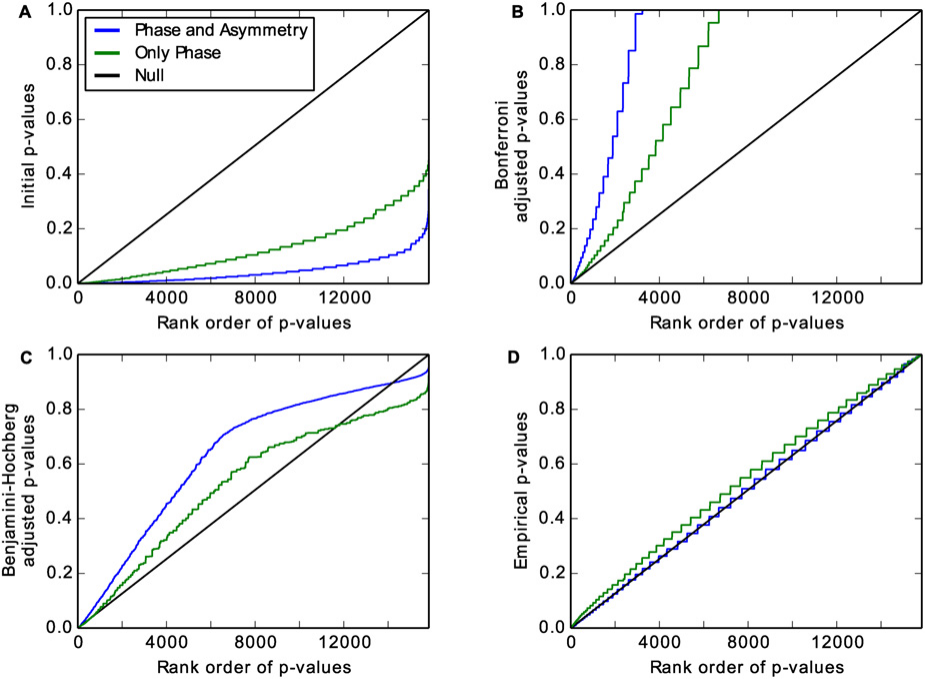
Empirical **p**-values are uniformly distributed for the null model of JTK_CYCLE. *P*-values versus their ranks from lowest (most significant) to highest (least significant) for JTK_CYCLE testing phases at 2 h intervals (green line) or phases and asymmetries at 2 h intervals (blue line) with time series consisting of Gaussian noise. Unbiased estimates should follow the black line (see text). (A) “Initial” *p*-values from JTK_CYCLE with multiple hypothesis testing underestimate the true *p*-values. (B) The Bonferroni correction results in *p*-values that are too high (less significant). (C) The Benjamini-Hochberg correction performs better than the Bonferroni correction but still results in *p*-values that are generally too high. (D) Empirical *p*-values that we calculate by permutation are close to uniformly distributed, as desired; their correspondence to the null model improves as the number of hypotheses tested increases.

It is important to note that *τ* (Eq. 1) is calculated for a specific reference time series, and thus JTK_CYCLE typically tests against a family of curves (e.g., to consider the possible phases of a waveform, as illustrated in Fig. 1B). It is thus necessary to account for multiple hypothesis testing across reference waveforms in assessing the significance of the results. Hughes *et al*. [26] employed the Bonferroni correction [34] in their original formulation and implementation of the method. This method is known to be conservative [34], and we illustrate this fact here explicitly for JTK_CYCLE (Fig. 2). These considerations motivate a new procedure for estimating the significance of the results, which we describe. We end this section by discussing the comparison of time series to reference waveforms (Fig. 1C) other than the cosine waveform that was used originally. Together, our improvements allow for the JTK_CYCLE method to include additional reference waveforms in its rhythm detection without compromising sensitivity and specificity.

#### Empirical *p*-values

By definition, a *p*-value is the likelihood of obtaining a test statistic equal to or more extreme than the value that is observed if the null hypothesis is true—it increases cumulatively as one progresses through a set of rank ordered test statistics. In the case of JTK_CYCLE, under the null hypothesis, time series values are independent (and generated by the same noise distribution) and so the rank ordering of time series values is random. For a dataset generated from this null model, the *p*-values should be uniformly distributed from 0 to 1, exclusive: the highest Kendall’s *τ* out of *N* tests should have a *p*-value of 1/(*N* + 1), the second highest test statistic has a *p*-value of 2/(*N* + 1), and the *i* ^*th*^ highest test statistic has a *p*-value of *i*/(*N* + 1) [35]. Restated, the *p*-values should be a linear function of the ranks (black lines in Fig. 2).

JTK_CYCLE computes the Kendall *τ* values for all the reference time series against the signal of interest and then performs a selection step for the lowest *p*-value (i.e., the highest *τ*), which we refer to here as the “initial” *p*-value. This procedure biases the *p*-values (the blue and green lines in Fig. 2A) such that they underestimate the true probability of obtaining test statistics by chance (the black line in Fig. 2A). The previous version of JTK_CYCLE corrects for underestimating the *p*-values with the Bonferroni correction, which controls the family-wide error rate (FWER) by multiplying the *p*-values by the number of hypothesis tests being performed. The FWER is the probability that there is at least one false positive for a given threshold. Therefore, a threshold of 0.01 means that there is a 1% chance that the list of time series with a Bonferroni adjusted *p*-value below 0.01 contains a false positive. This method, while rigorous, is overly conservative and overcompensates for the bias that comes from selecting the lowest *p*-value (blue and green lines in Fig. 2B). The likelihood of false positives is greatly reduced, but so is the likelihood of identifying true positives.

A common alternative to the Bonferroni correction is the Benjamini-Hochberg procedure [36], which seeks to control the false discovery rate (FDR). The FDR is the fraction of the time series that are identified as cycling that are false positive. For example, a Benjamini-Hochberg adjusted *p*-value cutoff of 0.05 means that 5% of the positives are false. This is a less stringent constraint than the FWER. In this procedure, the *p*-values are also multiplied by the number of hypotheses tested, as in the Bonferroni procedure. However, the *p*-values are additionally ordered from lowest to highest and then divided by their rank order (the lowest *p*-value has rank order 1, the second highest *p*-value has rank order 2, and so on). The *p*-values are also adjusted such that there is no change in ordering: if the originally lowest *p*-value is adjusted so that it is higher than the originally second lowest *p*-value, the lowest *p*-value takes the value of the adjusted second *p*-value so that the ordering is not violated. The same holds for the relationship between the second and the third lowest *p*-values and so forth. While the Benjamini-Hochberg procedure is a reasonable approach to multiple hypothesis testing in general, it does not account for the selection step in JTK_CYCLE; it still is thus overly conservative (Fig. 2C).

Consequently, we instead numerically compute the null distribution by applying the full JTK_CYCLE procedure to time series in which the values have random rank orders. Since we test a family of curves (e.g., spanning phases), we focus on positive correlations and compute one-sided *p*-values. In the present study, these “empirical” *p*-values are based on 2 × 10^6^ random time series and are nearly uniformly distributed, as desired (Fig. 2D). Though this empirical calculation is more computationally expensive than the application of the Bonferroni correction or the Benjamini-Hochberg correction, we show that empirically calculating the *p*-values results in better rhythmic detection and biological insight. We term this improved method *empirical* JTK_CYCLE, as we empirically calculate the *p*-values after selecting the highest *τ* value for each time series.

Below, we compare the Bonferroni adjusted *p*-values, Benjamini-Hochberg adjusted *p*-values, and empirical *p*-values directly. These have been adjusted on the basis of correcting for multiple hypothesis testing across different waveforms for a single time series. When we compare different time series to each other, we have to correct again for multiple hypothesis testing, this time across time series. To do this we use the Benjamini-Hochberg correction, as in the original implementation of the method [26]. When we refer to the Bonferroni, Benjamini-Hochberg, or empirical method, we refer to corrections across different waveforms for a given time series; all corrections across time series are with the Benjamini-Hochberg method. While the Bonferroni adjusted *p*-values, Benjamini-Hochberg adjusted *p*-values, and empirical *p*-values represent different quantities (the FWER, the FDR, and the *p*-values, respectively) they are all at least as conservative as the “true” *p*-values in the range that we are examining (compare blue and green lines with black lines in Figs. 2B, C, and D). This means that the FDRs that result from the Benjamini-Hochberg correction between time series are more conservative than the true FDRs.

#### Asymmetric waveforms

There is no *a priori* reason biological time series need be sinusoidal [37, 38], so it is of interest to test additional waveforms. In this regard, it is important to keep in mind that for JTK_CYCLE the rank order of the points in the reference matters, so we can represent a wide range of simple waveforms (e.g., Fig. 1C) by a triangle function with a specified separation between the maximum and the minimum. This allows us to avoid functionally defining an asymmetric cosine waveform. For the time series that we examine in this paper the difference is insignificant (S2 Fig.). We term the size of the interval from the maximum to the minimum the “asymmetry”, and we express the asymmetry here in units corresponding to a 24 h period. In this notation, a cosine has an asymmetry of 12 h, while a time series with an asymmetry of 8 h has a more rapid fall than rise (the values decrease over 8 h and increase over 16 h). The triangle reference waveforms have the same monotonicity as a cosine, and we keep the convention that the peak value corresponds to the phase of the time series.

To parse the effects of empirically calculating the *p*-values from those of including asymmetric waveforms, we test our form of JTK_CYCLE with and without asymmetric reference time series. In the former case, we denote searching over asymmetry values in steps of 2 h “by 2 h”, in steps of 4 h “by 4 h”, etc. We expect these additional waveforms to be more sensitive to asymmetric patterns of gene expression, resulting in discovery of additional rhythmic time series. It is important, however, to be cognizant of the fact that we are increasing the total number of hypotheses that we test, resulting in a greater need for the empirical correction procedure. Fig. 2 shows the different correction methods for the minimum *p*-values for JTK_CYCLE with searching over 12 phases (every 2 h, green line) or searching over 12 phases and 11 asymmetries (every 2 h, blue line). The added hypotheses for searching across asymmetries result in larger selection bias when choosing the highest *τ* value (Fig. 2A) as well as larger correction biases (Fig. 2B and C) than when only searching across phases. The empirical calculation of the *p*-values improves as the number of tests increases as well (Fig. 2), further justifying its use.

## Results

### Simulated data benchmarks

To assess the performance of our empirical form of JTK_CYCLE against the original form as well as other methods, we utilize two simulated datasets. We employ the first simulated dataset to understand the sensitivity of each method to different shapes of time series. It comprises four types of waveforms: sine, ramp (a triangle with maximum asymmetry), impulse, and step, as well as an equal number of time series consisting of Gaussian noise. We compare all the precision-recall curves for all the methods on these data via the area under the receiver operating characteristic (AUROC), a measure of the sensitivity and specificity of the rhythm detection methods that does not depend on the proportions of positives and negatives in the dataset. The second simulated dataset contains 10% rhythmic time series of triangle waveform with uniformly distributed phases and asymmetries and 90% time series consisting solely of Gaussian noise. We use it to further assess the importance of considering asymmetric waveforms, and we explore how multiple hypothesis correction impacts the results when the true positives represent a relatively small fraction of the simulated time series, as we expect to be the case in genome-wide studies.

#### F24, ANOVA, and JTK_CYCLE outperform other methods

**Fig 3:**
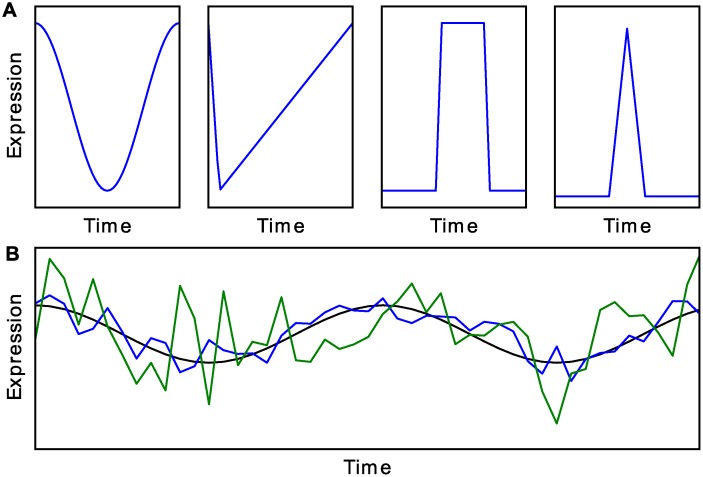
Examples of simulated data. (A) Different waveforms simulated with a 24 h period. From left to right, cosine, ramp, step, and impulse (width at half-max is 2 h). Waveforms in figure may not be to scale. (B) Cosine in black, with Gaussian noise with standard deviation of 25% (blue) or 50% (green) of amplitude.

To construct the first dataset described immediately above, for each of the four waveforms in Fig. 3A we generated 10,000 time series with uniformly distributed random phase shifts (always with a 24 h period) and added Gaussian noise to each point with a standard deviation of 25% or 50% of the total waveform amplitude, examples of which can be seen in Fig. 3B. We tested data with 4, 6, 8, or 12 evenly spaced points per 24 h period, and 1, 2, 3, or 4 replicates per time point (which is the equivalent of 1, 2, 3, or 4 periods per time series). At each spacing and replicate count we also generated 10,000 time series of Gaussian noise to serve as true negatives. The cyclohedron test, address reduction, stable persistence, and F24 are designed for single-replicate data, so we treated replicates as subsequent days of data, yielding multiple-period time series.

We scored each method by computing the area under the receiver operating characteristic curve (AUROC). The receiver operating characteristic (ROC) curve plots the true positive rate (TPR) as a function of the false positive rate (FPR) as the threshold for calling a time series as a positive is varied. The TPR and FPR are the fractions of the 10,000 simulated or Gaussian noise time series determined to be rhythmic at a threshold, respectively, and the threshold is varied over the entire range of false positive scores, such that the FPR ranges from 0 to 1. The AUROC is the integral of this curve; perfect classifiers have an AUROC of 1.0, while random classifiers have an AUROC of 0.5. An advantage of the AUROC as a metric is that it does not depend on the proportions of positives and negatives in the dataset because the TPR and FPR are calculated separately, i.e., they are normalized by the total number of positives and negatives, respectively. For stable persistence, the cyclohedron test, and address reduction, we calculated the AUROC from the test statistics themselves as opposed to the *p*-values, which we use for the latter three methods. Although the AUROC for JTK_CYCLE can be in principle be computed directly from the Kendall’s *τ* statistic, we include the multiple hypothesis testing correction because it impacts the TPR and FPR in practice; in particular, aggressive correction can lead to a loss of rank information because *p*-values must be less than or equal to 1.

**Fig 4:**
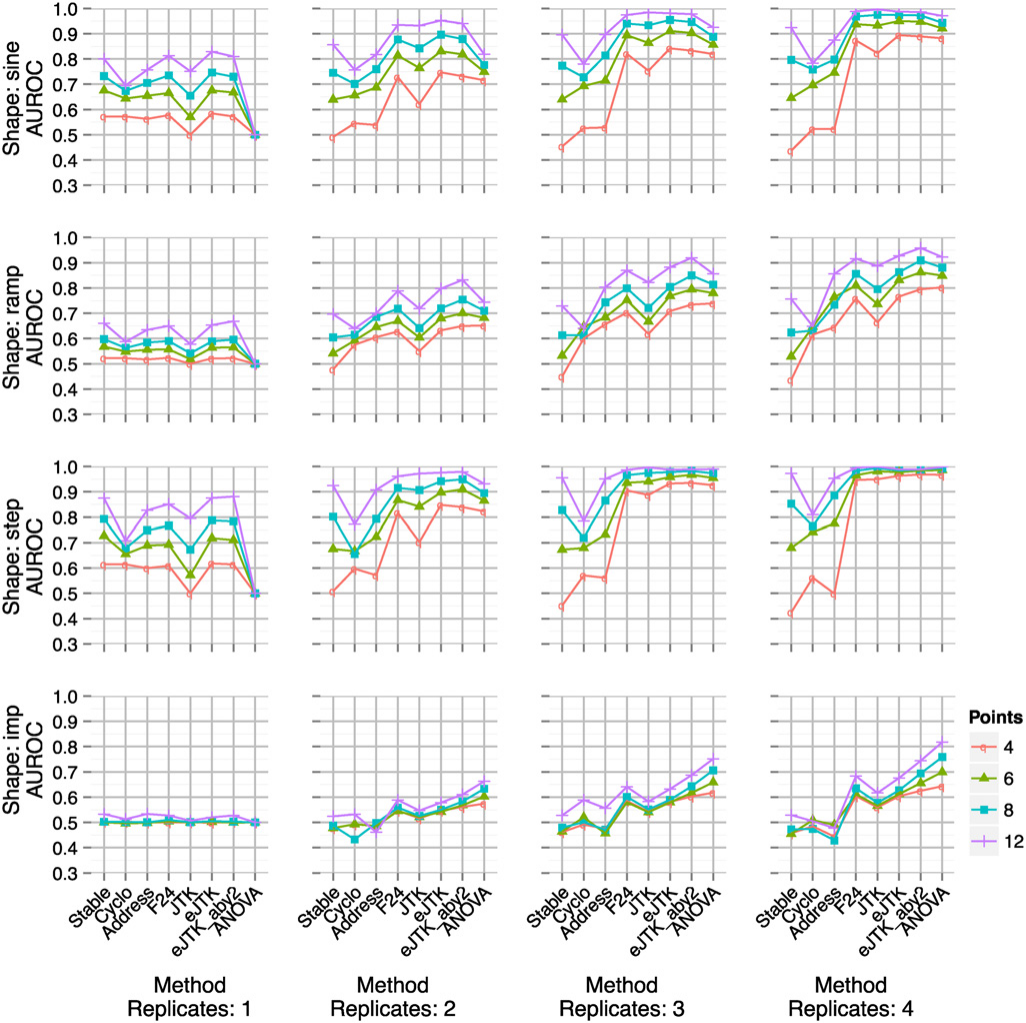
AUROCs for simulated data with 50% noise (standard deviation of Gaussian noise as a percent of amplitude). An AUROC value of 1 represents perfect discrimination between rhythmic and arrhythmic time series, and a value of 0.5 corresponds to random guessing. In each panel, the number of replicates increases from 1 to 4 replicates from left to right, and the number of sampled points per period is indicated by color. AUROC for single-replicate ANOVA (for which the method is undefined) is set at 0.5 exactly. Imp: impulse waveform, Cyclo: cyclohedron test, Address: address reduction, Stable: stable persistence, JTK: original JTK_CYCLE with Bonferroni correction, JTK_BH: JTK_CYCLE with Benjamini-Hochberg correction with symmetric triangle reference, eJTK: empirical JTK_CYCLE with symmetric triangle reference, JTK_BH_aby2: JTK_CYCLE with Benjamini-Hochberg correction and triangle references with asymmetries from 2 to 22 h by 2 h, eJTK_aby2: empirical JTK_CYCLE with triangle references with asymmetries from 2 to 22 h by 2 h.

The performance of the different methods at 50% noise can be seen in Fig. 4 (performance at 25% noise can be seen in S3 Fig.). ANOVA requires multiple measurements at each time point to determine the variance, so we define ANOVA to have an AUROC of 0.5 (performs no better than random guessing) when there is only one replicate. At 25% noise and high sampling rate, the cyclohedron test, address reduction, and stable persistence all perform roughly equivalent to the JTK_CYCLE methods, F24, and ANOVA. However, the former do noticeably worse than the latter at 50% noise. Empirical JTK_CYCLE out-performs original JTK_CYCLE, F24, and ANOVA for the sine and ramp waveforms, while ANOVA generally outperforms the other methods for the step and impulse waveforms.

While the empirical calculation approximates the null model well, it does not fully prevent multiple hypothesis testing from weakening the ability to identify rhythmic time series. Therefore, we do not sample phases and asymmetries more densely than the resolution of the data (e.g., if the experimental time points are spaced every 4 h, then we do not test phase values spaced every 2 h). We break this rule in Fig. 4 for the time series with 4–8 points for consistency of the figure. Sampling phases and asymmetries more densely than the resolution of the data needlessly reduces the power of our test, but does not affect the analysis in Fig. 4.

The JTK_CYCLE with Benjamini-Hochberg correction (JTK_BH) has AUROC values that are in between the AUROC values for the original JTK_CYCLE with Bonferroni correction (JTK) and empirical JTK_CYCLE. This is to be expected since the Benjamini-Hochberg method is more conservative than the empirical method but less conservative than the Bonferroni method. An additional detail is that the original JTK_CYCLE here uses a cosine as a reference waveform, in comparison to the triangle used by the other JTK_CYCLE methods. The methods that use the triangle waveform do not do significantly worse than the methods that use the cosine waveform in any of the cases, justifying the use of the triangle waveform for rhythm detection.

The cyclohedron test, address reduction, and stable persistence fail to improve as the number of replicates increases, and perform worse for low sampling rates. For example, a sine wave sampled at 4 time points per period for multiple periods has extrema at every other time point. Because cyclohedron test, address reduction, and stable persistence are essentially tests of monotonicity, they fail to detect the sparse periodic pattern in such data. In fact sparsely sampled data sometimes results in scores consistently lower than expected by chance, leading to the AUROC values less than 0.5 for these methods on some datasets in Fig. 4.

In summary, we find that all the methods tested can identify rhythmic expression patterns when the sampling density, replicate number, and signal-to-noise ratios are high. If data are sparse or noisy, however, method choice can significantly impact rhythm detection. In such cases, we find that ANOVA, F24, and JTK_CYCLE consistently better distinguish true and false positives. Empirical JTK_CYCLE outperforms ANOVA, F24, and original JTK_CYCLE for sine and ramp waveforms, but ANOVA performs better for impulse waveforms.

#### Increasing replicate number for a fixed number of total measurements improves sensitivity

**Fig 5:**
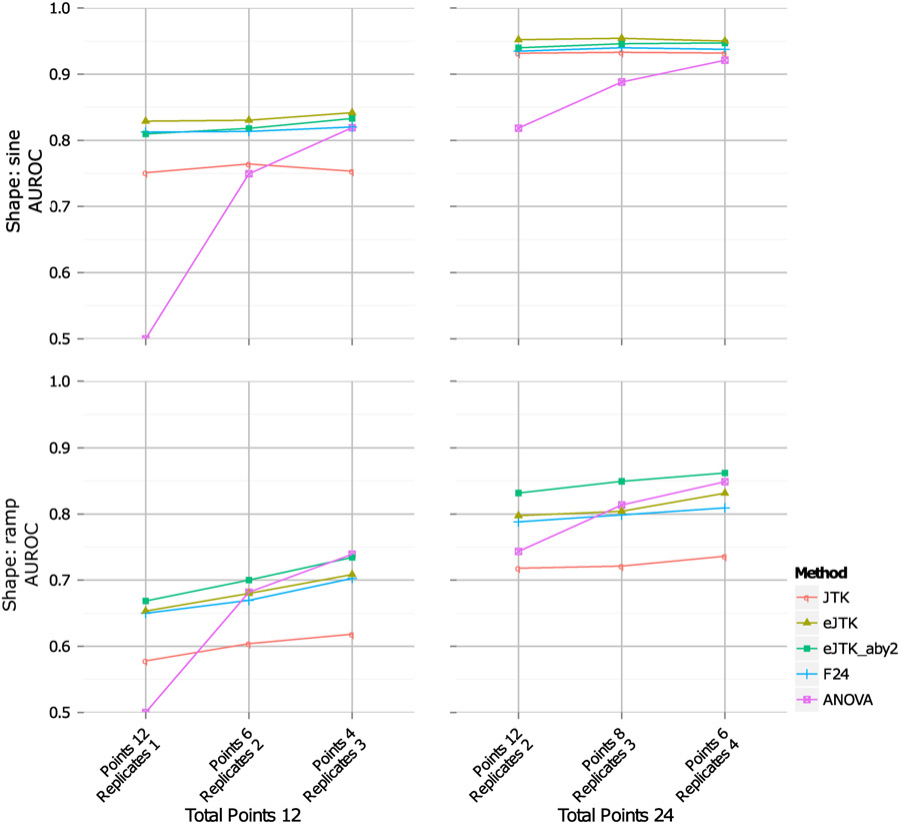
Higher numbers of replicates provide greater sensitivity compared to increased density of time points for the same number of samples. Results shown are AUROC values for sine and ramp simulated data with 50% noise (see S4 Fig. for additional waveforms and sample numbers). “Points” refers to the number of time points per period (“Points 12” refers to 12 points per period) and “Replicates” refers to the number of replicates per time series (“Replicates 2” refers to 2 samples per time point). Together, “Points 12 Replicates 2” refers to a time series that consists of 12 time points per period with 2 replicates per time point. Abbreviations are the same as in Fig. 4.

The total number of samples required for an experiment is the product of the number of time points and the number of replicates. Consequently, it is important to consider how best to apportion resources. To this end, in Fig. 5 we compare possible combinations of numbers of time points and replicates that give rise to 12 or 24 total samples (see S4 Fig. for additional waveforms and numbers of samples). For this comparison, we consider sampling at a density of 4 points per period to be the minimal requirement for the identification of rhythmicity. Furthermore, we focus on genome-wide experiments where the experimental design is such that there is no meaningful difference between data collected over multiple periods and data collected at the same sampling rate in replicate over a single period. This assumption does not hold for experiments that follow the response to a synchronization event or other perturbation because time points from successive periods are not equivalent.

To optimize the performance of ANOVA, it is best to maximize the number of replicates at the expense of the number of time points, which is not surprising given the importance of accurately estimating the variance in this test. For JTK_CYCLE and F24, the choice is less clear, but greater improvement is obtained with replicate increases in the case of the step and impulse waveforms (S4 Fig.). By contrast, original JTK_CYCLE performs slightly better for sinusoidal waveforms with higher numbers of time points, but empirical JTK_CYCLE does not. Overall, the results suggest that limited resources are better directed at increasing replicate numbers than the density of time points.

#### Interpolated pseudo-replicates improve ANOVA sensitivity

Given the importance of replicates in improving sensitivity, we also explored interpolating neighboring time points to create pseudo-replicates, which would double the number of time points in the data (S5 Fig.). However, this requires recomputing null distributions via MC sampling because the construction procedure introduces correlations between data points, resulting in *p*-value underestimates if not corrected. We found that the pseudo-replicates improved the performance mainly of ANOVA when the replicate number was low (e.g., 1 or 2); in particular, they allowed ANOVA to be applied and give good results for the single replicate case (S6 Fig.). We stress that 2 or more biological replicates should be obtained if at all possible, and we do not recommend using the pseudo-replicate approach if sufficient data are available.

#### Empirical JTK_CYCLE outperforms other methods after correcting for multiple hypothesis testing

Our second benchmark comprises 15,840 times series, which was chosen to allow equal numbers of time series with different phases and asymmetries. 10% of the time series were generated from a triangle waveform with noise added and 90% were generated entirely from Gaussian noise. This composition was chosen to be reflective of a genome-wide dataset. The rhythmic time series were 24 points long, with 2 periods, each with 12 time points. Here, we analyze two such datasets: one with only asymmetry of 12 h (analogous to a cosine) and one with a uniform sampling of possible asymmetries (by 2 h from 2 to 22 h). In both cases, phases (peak values) were uniformly distributed over the possible discrete values. We added Gaussian noise with a standard deviation of either 25% or 50% of the amplitude of the time series, as previously described. We tested these data against the empirical JTK_CYCLE method with various asymmetries as well as original JTK_CYCLE, ANOVA, F24, and Benjamini-Hochberg adjusted JTK_CYCLE with various asymmetries for comparison. In all cases the JTK_CYCLE methods used the triangle waveform as the reference waveform, as it was the waveform used to generate the data.

**Fig 6:**
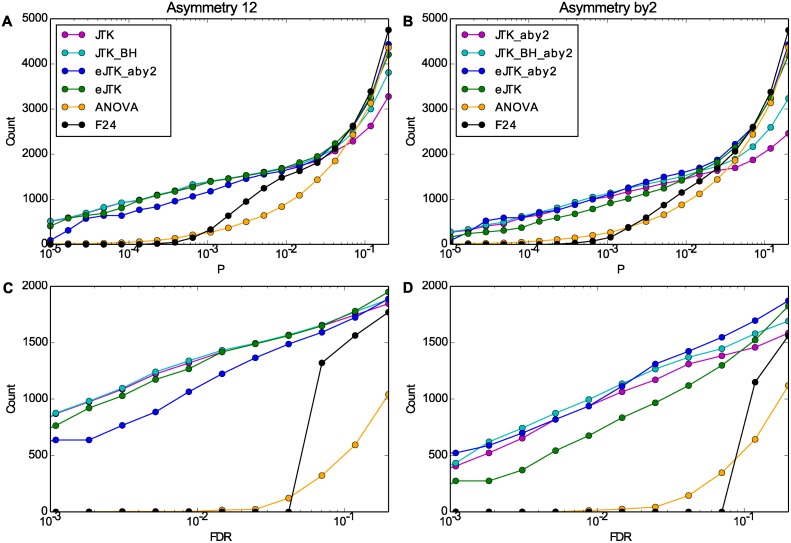
Empirical JTK_CYCLE outperforms the other methods in the presence and absence of asymmetric time series. Simulated data with rhythmic time series without asymmetry (left, A and C) or with evenly distributed asymmetry (right, B and D) were tested with different methods. The cumulative histograms are plotted before (A and B) and after (C and D) Benjamini-Hochberg multiple hypothesis correction across time series. The vertical axis shows the number of time series with a *p*-value (P) (A and B) or false discovery rate (FDR, the Benjamini-Hochberg adjusted *p*-value) (C and D) below or equal to a significance threshold, shown on the horizontal axis. Results shown are for the second simulated dataset with 25% noise, but the effects of Benjamini-Hochberg correction are significantly greater at 50% noise (not shown). The method abbreviations are the same as those in Fig. 4. The legends of A and B correspond to C and D, respectively. The rightmost point on the horizontal axis is 0.2.

We show cumulative histograms of the number of cycling time series identified for a given significance cutoff in Fig. 6. The methods shown yield comparable numbers for *p*-values less than 0.05, a reasonable threshold (Fig. 6A and B). However, in reporting total cycling numbers, it is important to correct for the fact that we are testing many time series (as opposed to testing many waveforms for a single time series, as previously). The *p*-values of empirical JTK_CYCLE are approximately uniformly distributed (Fig. 2D), as are those of ANOVA and F24, which satisfies the assumptions of the Benjamini-Hochberg correction [36], described above, so we use it for this purpose. We also apply the Benjamini-Hochberg correction to the original JTK_CYCLE with the intra-time series Bonferroni and Benjamini-Hochberg corrections discussed previously, which results in underestimates of the true FDR since their adjusted *p*-values are conservative (Fig. 2B and C).

In Figs. 6C and D, we see that the performance of the methods differs considerably when controlling for the false discovery rate (FDR). For these curves, the proportion of false positives identified as cycling matches the FDR. Specifically, the Benjamini-Hochberg correction (for many time series) penalizes methods with many *p*-values clustered at relatively high values (corresponding to a rapid rise toward the right of Figs. 6A and B, as for ANOVA and F24). Thus despite the fact that ANOVA and F24 perform comparably to JTK_CYCLE in the AUROC analysis (Fig. 4), their *p*-values provide less discrimination between time series, and thus they provide less sensitivity for a given FDR. In addition to looking at AUROC scores in Fig. 4 and time series identification in Figs. 6C and D, we also computed the Matthews Correlation Coefficient [39], which quantifies the quality of a binary classification. A score of 1 indicates that a method correctly identified all true positives and true negatives, while a score of −1 indicates that a method yielded all false positives and false negatives. S7 Fig. shows that the JTK_CYCLE methods have higher-quality classification ability than F24 and ANOVA for these simulated data. Furthermore, the figure shows that empirical JTK_CYCLE with asymmetry search performs equally well with and without asymmetric time series, whereas the JTK_CYCLE methods without asymmetry search perform worse when the dataset includes asymmetric time series.

Therefore, in terms of genome-wide studies, empirical JTK_CYCLE with asymmetric waveforms is the method of choice for identifying rhythmic genes. S8 Fig. examines how the inclusion of different asymmetries affects rhythm detection.

### Microarray metadataset

Keegan *et al*. [27] previously assembled a metadataset comprised of data from four DNA microarray studies of *Drosophila melanogaster* under light-dark (LD) conditions (from Ceriani [40], Claridge-Chang [18], Lin [41], and Ueda [42]). We do not include a fifth dataset from that study [15], because it was limited to dark-dark (DD) conditions. Here, we discuss issues that arise from merging data from different laboratories and use the resulting metadataset to test the methods. We find that empirical JTK_CYCLE with asymmetry search identifies a larger number of rhythmic genes and, in turn, enriched annotations among those genes, such as oxidation reduction, glutathione metabolism, and alternative splicing.

#### Z-score-based procedure for preparing the metadataset

**Fig 7:**
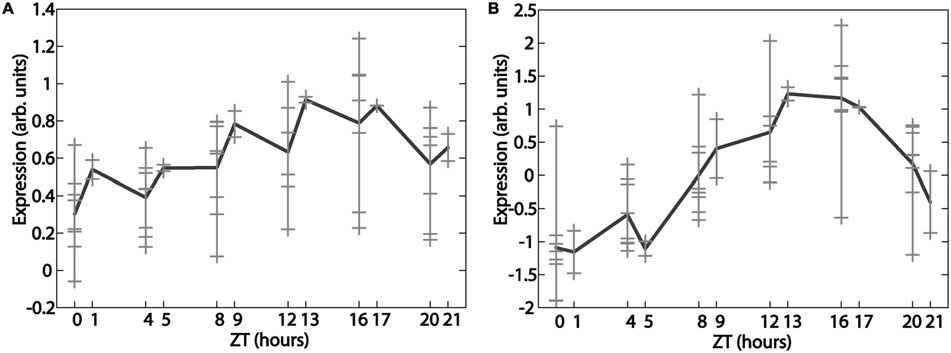
Z-score normalization allows combining of time series from different datasets into smooth time series. *Pdp1* gene expression from metadata before (A) and after (B) Z-score normalization. Light gray crosses indicate individual replicates, and the black curve is the mean.

All of the measurements in the contributing studies are at intervals of 4 h. Time points for circadian LD time series are referenced as zeitgeber time points (ZT); the beginning of the light period is ZT0. Under 12 hours of light and 12 hours of dark, ZT24 is the equivalent of ZT0. Three studies sampled at ZT0, 4, 8, 12, 16, and 20, and the fourth (Ueda [42]) sampled at ZT1, 5, 9, 13, 17, and 21. We found that the differences in sampling protocols, together with variations from one laboratory to another, consistently gave rise to a jagged structure in the time series of known cycling genes (Fig. 7A). Microarray-wide normalization techniques such as quantile normalization were unable to produce curves consistent with independently measured profiles. Instead, we found that the best approach was to convert the values in each time series to Z-scores—i.e., for each gene in each dataset, we subtract its mean expression level and divide by its standard deviation. Then we pool the Z-scores to generate the metadataset. Fig. 7 illustrates the effect of the processing step on *Pdp1*, a known cycling gene. This method is equivalent to treating measurements from the same zeitgeber time point as replicates. For probes that corresponded to the same gene, we chose the probe with the highest mean expression value to use in the analysis. This reduced 14,010 probes to 11,625 genes.

The metadata has many places where the values are not available (NA). To prevent the need to recalculate the null distribution for every pattern of NAs in the data for empirical JTK_CYCLE (there were 5005 unique NA permutations in the data), the NAs were replaced by random noise drawn from a Gaussian distribution with mean and standard deviation that match those of the data on the whole. While this adds noise to the time series, it should not have a large effect given that each time series has 57 points. To mitigate the impact of this procedure on our study, however, time series that had more then half their points as NA were discarded from the dataset, leaving 9,313 out of 11,625 genes. We consistently used the dataset resulting from these preprocessing steps for all our analysis to ensure that comparisons between methods were fair; where comparisons with and without NA substitution were possible, we found that NA substitution led to slight increases in cycling numbers in all cases except ANOVA (114 vs. 101). However, these differences did not change any of the ontological results (discussed below).

#### Analysis of microarray metadataset

To evaluate the methods against genes for which we know the rhythmicity *a priori*, we compared the *p*-values for 6 positive examples (*per*, *tim*, *vri*, *Pdp1*, *cry*, and *Clk*) and 4 negative examples (*cam*, *RpL32*, *cyc*, and *dco*). S9 Fig. shows the performance of the different methods for the known positive and negative examples. Stable persistence, the cyclohedron test, and address reduction all have false negatives. The JTK_CYCLE methods, ANOVA, and F24, however, detect all of the known cycling genes and none of the non-cycling genes as rhythmic.

**Fig 8:**
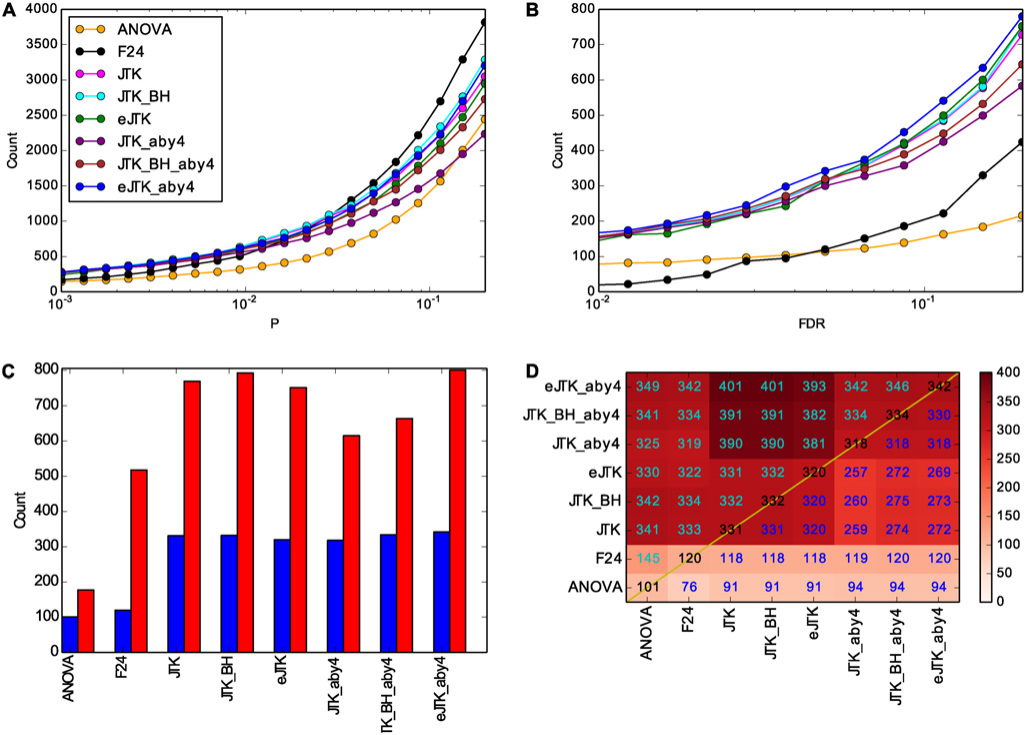
Empirical JTK_CYCLE with asymmetry search of 4 h (eJTK_aby4) identifies more genes than ANOVA, F24, and the other JTK_CYCLE methods. (A) The vertical axis shows the number of genes with a *p*-value below or equal to the horizontal axis for the methods indicated. The rightmost point on the horizontal axis is 0.2. (B) The Benjamini-Hochberg correction for testing multiple genes impacts the relative performance of the different methods. The rightmost point on the horizontal axis is 0.2. The colors are the same as in A. (C) The number of genes with Benjamini-Hochberg adjusted *p*-values below 0.05 (blue) and below 0.20 (red) with the different methods is shown. (D) A comparison of the intersection (below the diagonal) and union (above the diagonal) of genes identified as rhythmic with Benjamini-Hochberg adjusted *p*-values less than 0.05 for the different methods. JTK: the original JTK_CYCLE method with Bonferroni correction. JTK_BH: the JTK_CYCLE method with Benjamini-Hochberg correction. eJTK: the JTK_CYCLE method with empirical calculation of the *p*-values. “_aby4” refers to an asymmetry search every 4 h (at 4, 8, 12, 16, and 20 h).

Having again established F24, ANOVA, and JTK_CYCLE as the better methods, we now apply them to the full dataset (Fig. 8). As in Fig. 6, the Benjamini-Hochberg correction decreases the sensitivity of ANOVA and F24 relative to JTK_CYCLE for a given FDR (compare Fig. 8A with Fig. 8B). Choosing a Benjamini-Hochberg adjusted *p*-value cutoff of 0.05 (i.e., 5%), the number of genes and overlap between methods can be seen in Figs. 8C and D. All the JTK_CYCLE methods outperform F24 and ANOVA. Empirical JTK_CYCLE with asymmetry search by 4 h (eJTK_aby4) identified the most genes, showing a clear improvement over the Bonferroni (JTK) and Benjamini-Hochberg (JTK_BH) methods with asymmetry search by 4 h (aby4); eJTK_aby4 also identified more cycling genes than methods without asymmetry search, and the genes were distinct.

Interestingly, among the JTK_CYCLE methods without asymmetry search the Bonferroni and Benjamini-Hochberg methods identified more genes than the empirical method did. For JTK_CYCLE without asymmetry search, there were only 6 hypotheses tested per gene time series (for each of the 6 phases searched), for which the Bonferroni and Benjamini-Hochberg correction across waveforms is very slight. For JTK_CYCLE with asymmetry search every 4 h, the number of hypotheses tested becomes 30, for 6 different phases paired with 5 different asymmetries, which results in a more stringent correction by the Bonferroni and Benjamini-Hochberg methods. As experimental sampling rates and sampling densities enable more extensive searching of phases, periods, and asymmetries, we expect the advantage for empirical JTK_CYCLE relative to the original formulation to grow because the Bonferroni correction strongly penalizes adding hypothesis tests. Provided that sufficient permutations are performed, empirical JTK_CYCLE provides the more robust identification of rhythmic genes. Another way of viewing this difference between the inclusion and exclusion of asymmetry search is by examining the distributions of the Bonferroni-adjusted *p*-values against the empirical *p*-values, as in S10 Fig. With asymmetry search, the empirical *p*-values are significantly lower than the Bonferroni-adjusted *p*-values, a pattern that is less pronounced without asymmetry search.

We examine the effect of searching multiple asymmetries with empirical JTK_CYCLE further in S11 Fig. Searching for rhythmic genes with asymmetries of 8 and 16 h alone yielded 4 more genes than searching for rhythmic genes with asymmetries of 4, 8, 12, 16, and 20 h, with an overlap of 293 genes and approximately 50 genes each that were separately called rhythmic by each method. Comparing the two sets of cycling genes in S12 Fig., we find that searching by 4 h excludes genes with asymmetries 8 to 16 h that are barely below the adjusted *p*-value of 0.05 (upper left quadrant), while searching at asymmetries of 8 and 16 h excludes genes that have extreme asymmetries.

We also examined how our results depended on using a triangle vs. a cosine for the reference waveform. Figs. S13 and S14 show that there is no substantial difference in genes identified as cycling or in ontological results (discussed below). This can be attributed to the fact that across many time points (57 in the case of the metadataset), the differences between the cosine and triangle waveform are slight (S2 Fig.).

#### Comparison with Keegan *et al*

We compared our results to those of Keegan *et al*. [27], an earlier analysis of this metadataset. There were two main differences in the way we constructed the dataset: we excluded time series that had more than half their values as NA, and we excluded the dark-dark (DD) McDonald dataset, as discussed above. Of the 200 genes identified as cycling by Keegan *et al*., 169 remained after pre-processing to remove time series with more than half of their values as NAs. Of those 169, 111 had Benjamini-Hochberg adjusted *p*-values less than 0.05 for the empirical JTK_CYCLE with asymmetry search by 4 h (eJTK_aby4). 58 genes that were identified as cycling by Keegan *et al*. were not identified by eJTK_aby4. S15 Fig. compares the cycling genes identified by Keegan *et al*. with the cycling genes identified by eJTK_aby4. Keegan *et al*. identified genes as cycling primarily on the basis of scoring well (*p* < 0.05) on several tests following pre-screening by ANOVA. S15A Fig. shows a comparison of the number of tests passed after the ANOVA pre-screening with the Benjamini-Hochberg adjusted *p*-value from eJTK_aby4. While there appears to be a weak relation between the number of tests passed and the *p*-value, there is not a clear pattern that would enable one to predict the cycling genes common to both Keegan *et al*. and eJTK_by4. S15B Fig. shows the maximum amplitude measurements (after Z-scoring) for the genes identified as cycling by Keegan *et al*., which are organized by whether they are identified as cycling by eJTK_aby4 as well. The genes identified by Keegan *et al*. but not by eJTK_aby4 tend to have larger maximum amplitudes than the ones identified by both. The ANOVA pre-screening in Keegan *et al*. can account for this difference; our results with empirical JTK_CYCLE suggest that there are many cycling genes with lower amplitudes. S15C Fig. shows the asymmetries of the genes identified by Keegan *et al*. as cycling, as determined by eJTK_aby4. A large number of genes identified by Keegan *et al*., but not by eJTK_aby4, have asymmetry of 16 h. The bias in the earlier study may reflect the fact that one of the tests that Keegan *et al*. employs is based on correlation with the gene *per*, which has an asymmetry of 16 h. More generally, Keegan *et al*. fail to identify 231 genes as cycling that eJTK_aby4 identifies with Benjamini-Hochberg adjusted *p*-values below 0.05. Of these 231, 82 have Benjamini-Hochberg adjusted *p*-values below 0.01, 65 have values below 0.005, and 16 have values below 0.001.

#### Comparison with Wijnen *et al*

In addition to comparing our results to those of Keegan *et al*., we also compared our results to those of Wijnen *et al*. [32], who identified 336 genes as rhythmic using an F24-based method. Again, we excluded time series that had more than half their values as NA, and we excluded the dark-dark (DD) McDonald dataset. S16 Fig. shows a comparison of the genes that are identified as rhythmic by eJTK_aby4, Keegan *et al*., and Wijnen *et al*. Whereas 31 genes that Keegan *et al*. identified as rhythmic were removed by the empirical JTK_CYCLE analysis pre-processing, 57 genes that were identified by Wijnen *et al*. were removed by the empirical JTK_CYCLE analysis pre-processing due to more than half their time points being NA. These genes are “unassigned” in S16A Fig. because an asymmetry estimate is not available. Wijnen *et al*. and eJTK_aby4 jointly identified 120 genes as rhythmic, of which Keegan *et al*. identified 59 as well. Wijnen *et al*. uniquely identified 177 genes as rhythmic, whereas eJTK_aby4 uniquely identified 167 genes. A comparison of the asymmetry distributions for all the genes (S16A Fig.) shows that they are similar for eJTK_aby4 and Wijnen *et al*.

#### Validation with dataset-independent literature citations

As a first step toward validation the new genes that eJTK_aby4 exclusively identified as rhythmic (i.e., those genes not previously identified by Keegan *et al*. or Wijnen *et al*.), we examined the literature for references that independently suggest that these genes are cycling. Specifically, for each gene, we identified the references in FlyBase (http://flybase.org) that mention the gene. Of those references, those that have the term “circadian” in their title or abstract were identified. S16B Fig. shows the distribution of genes based on their citation in FlyBase by a “circadian” paper, by the original five dataset papers [15, 18, 40–42], or by neither. Genes identified by “circadian” papers but not by the original five dataset papers represent further validation that the genes that we select as rhythmic are related to circadian processes.

Among these references, there were some that discussed several genes. Kadener *et al*. [43] assayed for genes regulated by the gene *Clk* and referenced 6 of the genes not mentioned by the original five papers out of a total of 32 genes, which has less than 1.6% probability of occurring by chance (Fisher’s Exact Test unadjusted *p*<0.016 [44]). One gene referenced by Kadener *et al*. as well as Abruzzi *et al*., who also assayed for genes regulated by *Clk*, is *cabut* (*cbt, CG4427, FBgn0043364*, referred to as EP2237 by Kadener *et al*.). The gene *cbt* was previously unidentified as having rhythmic expression. The average time series from the metadata can be seen in S17 Fig. The gene *cbt* is a metal-ion binding transcription factor downstream of the JNK cascade and is involved in morphogenesis [45–48]. It has an asymmetry of 4 h, potentially explaining why it was missed by previous methods but identified by eJTK_aby4. Abruzzi *et al*. [49] also discuss another *Clk*-regulated gene that was uniquely identified by eJTK_aby4 as rhythmic, *twins* (*tws, CG6235, FBgn0004889*), seen in S17 Fig. It has an asymmetry of 20 h, which explains how, like *cbt*, it could have been missed by previous methods. These genes, though previously unidentified as rhythmic, are strong candidates for having roles in circadian regulation and processes based on our identification of them as rhythmic and the work of Kadener *et al*. and Abruzzi *et al*. This warrants further experimental studies of these genes in a circadian context as well as the other genes that we have identified.

The gene *cbt* is also referenced by another study that discusses several genes identified as rhythmic by eJTK_aby4. Fujikawa *et al*. [50] identified 114 genes that are up-regulated and down-regulated in the head of *D. melanogaster* following 24 h of starvation. 16 of these genes are not mentioned by the original five papers but are identified as rhythmic by eJTK_aby4, which has less than 0.3% probability of occurring by chance (Fisher’s Exact Test unadjusted *p*<0.003). Fujikawa *et al*. refer to several genes from the circadian dataset papers that also appear in their lists of differentially expressed genes, but they do not associate rhythmic behavior with all the genes that they describe. In addition to the gene *cbt*, Fujikawa *et al*. reference other genes that were previously unidentified as rhythmic: *Esterase-Q* (*Est-Q, CG7529, FBgn0037090*) and *1, 4-Alpha-Glucan Branching Enzyme* (*AGBE, CG33138, FBgn0053138*). Both have asymmetries of 16 h, which is also outside the range of standard symmetric-waveform detection (S17 Fig.). The identification of these genes as rhythmic reinforces the connection between metabolism and circadian regulation and indicates other potential areas of experimental exploration.

To further understand the relationship between circadian regulation that we see in the genes eJTK_aby4 has identified as rhythmic and biological processes, we examined the enrichment of functional annotations in the identified genes.

#### Functional classification of cycling genes

**Fig 9:**
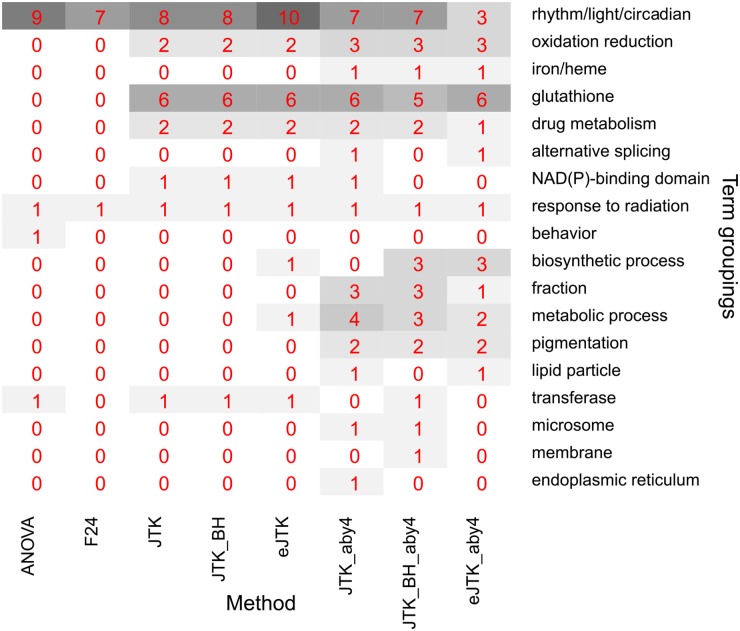
Manual grouping of annotation terms identified as enriched by DAVID. The number of annotation terms enriched in the genes with Benjamini-Hochberg adjusted *p*-values less than 0.05 that are identified by each method are shown in grey shading and red numbers. Annotation terms were enriched with Benjamini-Hochberg adjusted *p*-values below 0.05 as identified by the DAVID web tool [51, 52]. Abbreviations are the same as in Fig. 8.

We used DAVID [51, 52] to analyze the ontological enrichment of the genes contributing to Fig. 8 separately for each rhythm detection method. Because many of the annotation terms are obviously related (e.g., “oxidoreductase” and “oxidation reduction”), we manually grouped them. The grouped terms enriched with Benjamini-Hochberg adjusted *p*-values less than 0.05 for the different methods can be seen in Fig. 9. Genes that are identified as rhythmic by F24 and ANOVA are enriched in the fewest terms. They are mainly in rhythm/light/circadian categories, corroborating the selection of these genes as cycling. The JTK_CYCLE methods without asymmetry search identify sets of genes that are enriched for different terms in addition to the rhythm/light/circadian ones found by ANOVA and F24, such as glutathione and oxidation reduction annotation terms. The JTK_CYCLE methods with asymmetry search identify sets of genes enriched in the most terms of all the methods. The original JTK_CYCLE method with Bonferroni correction and empirical JTK_CYCLE method identify sets of enriched genes known to have alternative splice forms of their RNA; JTK_CYCLE with the Benjamini-Hochberg correction and empirical JTK_CYCLE identify sets of genes that are enriched for genes involved in biosynthetic pathways.

**Fig 10:**
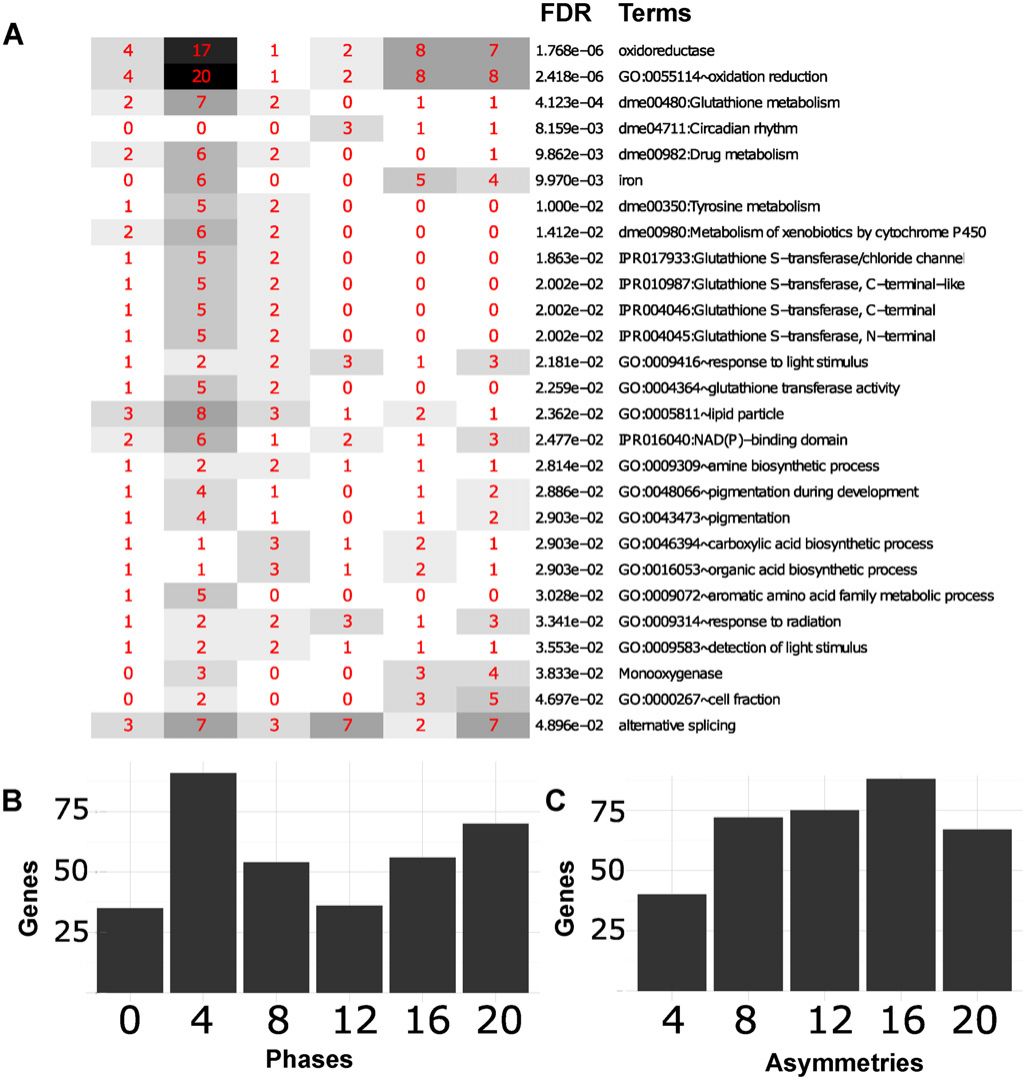
Annotation terms identified by DAVID as enriched for rhythmic genes. Rhythmic genes shown are those that are identified with eJTK_aby4 with a Benjamini-Hochberg adjusted *p*-value less than 0.05. The terms shown are those identified by the DAVID web tool [51, 52] as enriched with a Benjamini-Hochberg adjusted *p*-value less than 0.05. (A) The individual annotation terms are shown with their adjusted *p*-values and phase distributions. The red numbers refer to the number of genes in that annotation term with that phase. The horizontal axis of A is the same as that of B. (B) Total phase distribution of the cycling genes. (C) Total asymmetry distribution of the cycling genes.

Because eJTK_aby4 captures all the annotation terms of interest, we focus on its results for the remainder of this section. The individual annotation terms that are enriched in the rhythmic genes found by eJTK_aby4 can be seen with their adjusted *p*-values and phase distributions in Fig. 10A. Fig. 10B shows the total phase distribution of the genes, and Fig. 10C shows the total asymmetry distribution. Functionally, these genes fall into several annotation categories, each of which we discuss in turn.

Many of the rhythmic genes involved in glutathione metabolism are also involved in drug metabolism. The peak expressions of these genes are focused around ZT4, and these genes mainly have asymmetries close to 12 h (Figs. 10A and S18). Glutathione and drug metabolism are known to be circadian [53–55]; possible links to aging are suggested by the role of glutathione metabolism in clearing reactive oxygen species [56]. Other oxidation-reduction related terms peak at either ZT4 or ZT16-20 (Figs. 10A and S19). These genes have a broader distribution of asymmetries, with several with extreme values of 4 or 20 h.

A subset of the genes involved with oxidation-reduction are also associated with iron and have a bimodal distribution of phases, with peaks at ZT4 and ZT16-20 (Fig. 10A). Various iron-related genes have been implicated as important in circadian rhythms. Recent studies, however, have only looked at the effect of iron-related genes on whole organism activity, or on particular circadian genes, such as *per* or *tim* [57, 58]. These studies have shown that individual iron-related genes affect circadian rhythms. To our knowledge, no studies to date have found as many iron-related genes displaying rhythmic behavior as we have described here.

Genes that have multiple protein forms due to alternative splicing peak at times that are evenly distributed throughout the day (Figs. 10A and S20). They have a broad distribution of asymmetries as well, with several genes with extreme values of 4 h and 20 h, such as *tws*, the newly discovered cycling gene previously mentioned as having an asymmetry of 20 h and as a regulatory target of *Clk*. The existence of these alternatively spliced genes with extreme asymmetries explains why “alternative splicing” was only found to be enriched in the genes identified as rhythmic by methods searching for asymmetric waveforms. Alternative splicing has been found to be important in circadian rhythms in *Drosophila* as well as in other species. Most studies, however, have focused on specific experimental findings that discovered particular genes that modulate circadian rhythms [59, 60]. No studies exist that have found that so many genes with alternative splicing are rhythmic.

## Discussion

In this paper, we compare several rhythm detection methods. These approaches are general and can be applied to detecting periodic behavior in a wide range of contexts, but we focus on time series representative of genome-wide expression data. Deckard *et al*. [28] recently reviewed a number of earlier studies of rhythm detection methods and selected four algorithms for comparison (de Lichtenberg, Lomb-Scargle, JTK_CYCLE, and persistent homology) based on their mathematical properties and applicability to genome-wide expression data. They test the methods with simulated data and experimental data for the metabolic cycle in yeast, circadian rhythms in the mouse, and the root clock in the flowering plant *Arabidopsis thaliana* (see [28] for references). They find that there is no all-around best method and construct a decision tree for picking an algorithm based on the expected nature of the data. For increasing noise and decreasing sampling rate, they favor JTK_CYCLE and Lomb-Scargle, a Fourier-like method. These recommendations are consistent with our own findings that JTK_CYCLE and F24 were consistently more accurate than the monotonicity tests (represented by persistent homology in [28]) and justifies our focus on improving JTK_CYCLE.

Also recently, Zielinski *et al*. [29] reviewed six different Fourier-like methods for their ability to estimate periods in periodic time series. They focus on time series that are well-sampled (36 and 72 time points were the lowest-sampled time series they examined, 720 was among the highest), as might be obtained from tracking a luminescence reporter for a single gene. The fundamentally different nature of their data from ours highlights the fact that it is important to match the computational tool with the task of interest. Zielinski *et al*. [29] seek precise period determination for genes already known to cycle. By contrast, here we focus on discovering rhythmic time series that represent only a fraction of a genome-wide dataset. JTK_CYCLE can provide estimates of a periodic time series’ phase, period, and asymmetry, but it resolves these parameters only to the level of the sampling (or search) depth. It is likely that there is a tradeoff between robust separation of rhythmic and arrhythmic time series and precise estimation of the cycling parameters; presently, no algorithm achieves both these goals simultaneously.

Zielinski *et al*. [29], as well as earlier studies [37, 38], note that biological oscillations are expected to have asymmetric patterns of expression. Thaben and Westermark recently published one approach to this problem [30]. Their method, RAIN, employs the Mann-Whitney U test [61] between different time points to look for a rising pattern followed by a falling pattern. They show that RAIN outperforms the original JTK_CYCLE method as well as a cosine-fitting method [62] for simulated data consisting of sinusoidal and ramp waveforms. They also analyze genomic and proteomic data for the mouse liver. Their work reinforces our finding that searching for asymmetric waveforms can produce better rhythm detection sensitivity and validates our efforts. Thaben and Westermark suggest that modifying JTK_CYCLE to allow for asymmetric waveforms would provide a useful complement to their approach. In particular, in contrast to RAIN, JTK_CYCLE can search for specific waveforms, including arbitrary shapes with multiple peaks. We meet that need here.

We were able to expand JTK_CYCLE to search for asymmetric waveforms without degrading sensitivity because we empirically calculate *p*-values, which yields much more accurate significance estimates than the Bonferroni correction employed in the original formulation of JTK_CYCLE [26]. Our analysis of two different simulated datasets and the fly head metadataset clearly shows the importance of accurate significance estimates. As sequencing costs continue to decrease, we expect sampling density to increase. This trend should favor use of empirical JTK_CYCLE over alternative means of correcting for multiple hypothesis testing because the increase in data will enable more phases, asymmetries, and periods to be examined. Our analysis shows that certain gene ontologies have many genes with highly asymmetric patterns of expression. It will be interesting to determine the prevalence of different waveforms in additional biological datasets and to understand how their features depend quantitatively on genotype, tissue, and environmental conditions.

### Conclusions

In this paper, we compare methods for detecting rhythmic time series in genome-wide expression data. With regard to experimental design, we find that increasing the number of replicates is more important than increasing the sampling density for achieving greater sensitivity. A key aspect of our study is that we improve the estimation of *p*-values in JTK_CYCLE. This enables control of the false discovery rate and testing waveforms beyond sinusoidal ones. For both simulated data and a circadian metadataset [27] the resulting empirical JTK_CYCLE with asymmetry search exhibits the greatest sensitivity among the methods that we evaluated. The annotation terms that are enriched among the genes that we identify as cycling include rhythm/light/circadian, glutathione/drug metabolism, oxidation-reduction, iron metabolism, and alternative splicing. These findings are consistent with known circadian biology but also suggest new investigations.

## Supporting Information

S1 Fig Evaluation of Gamma distribution modeling for the F24 null distribution. The time series used for this example was a 24 h sine wave sampled every 2 h for 1 period (no replicates); noise was added at 25% of the amplitude. (A) Convergence of the mean and variance estimates, used to parameterize the Gamma distribution, as a function of the number of permutations performed, for testing the 24 h period (blue curve in A; convergence for 4 h and 48 h periods were similar, data not shown). (B) The cumulative distributions obtained by random permutation fit to the Gamma distribution, as shown by their proximity to the diagonal (black). Shown are fits for testing a 24 h period, plus a 4 h period and a 48 h period (i.e., F4 and F48). For these fits, 100 permutations were used.
(EPS)

S2 Fig The triangle waveform is highly correlated with the cosine waveform. The correlation between triangle and cosine waveforms are compared for time series of different lengths for three different correlation metrics: Pearson, Spearman, and Kendall. Correlations can range from −1 (completely anti-correlated) to +1 (completely correlated).
(EPS)

S3 Fig AUROCs for simulated data with 25% noise (standard deviation of Gaussian noise as a percent of amplitude). Layout and abbreviations are the same as in Fig. 4.
(EPS)

S4 Fig Full set of comparisons used to evaluate the trade-off between increased numbers of replicates and increased densities of time points per period. Layout and abbreviations are the same as in Fig. 5.
(EPS)

S5 Fig Interpolation scheme for increasing replicate counts. (A) A pseudo-replicate (⊙) for time *t*_*i*_ (indicated by the arrow) is obtained by linearly interpolating between time points *t*_*i*−1_ and *t*_*i*+1_ (dashed line). (B) Repeating this procedure for each time point (modulo 24 h) generates a new time series (⊙ symbols).
(EPS)

S6 Fig Interpolating the data points to generate pseudo-replicates improves AUROCs when the number of actual replicates is low. We compare performance with (Interp) and without (Normal) pseudo-replicates for the first simulated dataset with 50% noise.
(EPS)

S7 Fig Matthews Correlation Coefficient shows that JTK_CYCLE methods outperform ANOVA and F24 in the presence and absence of asymmetric time series. Simulated data with rhythmic time series without asymmetry (A) or with evenly distributed asymmetry (B) was tested with different methods. The vertical axis shows the Matthews Correlation Coefficients (MCC) [39] for different Benjamini-Hochberg adjusted *p*-value cutoffs (FDR) along the x-axis. These data are with 25% noise, but the effects of Benjamini-Hochberg correction are significantly greater at 50% noise (not shown). The method abbreviations are the same as those in Fig. 4.
(EPS)

S8 Fig Searching for asymmetric waveforms is detrimental if none are present, but is otherwise advantageous. Simulated data with rhythmic time series without asymmetry (left, A and C) or with evenly distributed asymmetry (right, B and D) was tested with different asymmetries. The cumulative histograms are plotted before (A and B) and after (C and D) Benjamini-Hochberg multiple hypothesis correction across time series. The vertical axis shows the number of genes with a *p*-value (P) (A and B) or false discovery rate (FDR, the Benjamini-Hochberg adjusted *p*-value) (C and D) below or equal to a significance threshold, shown on the x-axis. These data are with 25% noise, but the effects of Benjamini-Hochberg correction are significantly greater at 50% noise (not shown). The legend in A applies to B, C, and D as well as A. The rightmost point on the horizontal axis is 0.2. eJTK_aby2: asymmetries sampled every 2 h, from 2 h to 22 h, eJTK_aby4: asymmetries sampled every 4 h, from 4 h to 20 h, eJTK_a04-12-20: asymmetries sampled at 4 h, 12 h and 20 h, eJTK_a08-16: asymmetries sampled at 8 h and 16 h, eJTK: no asymmetry (i.e. asymmetry of 12 h, equivalent to a cosine).
(EPS)

S9 Fig Metadata results for known positive and negative examples. The positive examples are known cycling genes *per*, *tim*, *vri*, *Pdp1*, *cry*, and *Clk*. The negative examples are known non-cycling genes *cam*, *RpL32*, *cyc*, and *dco*. As plotted, large values for the positive examples and small values for the negative examples are desirable. The magenta line marks a *p*-value of 0.05 (−log_10_ 0.05 = 1.3). Since 2 × 10^6^ permutations were used to generate the empirical JTK_CYCLE *p*-values, they cannot be lower than 5 × 10^−7^. Abbreviations are the same as in Fig. 8.
(EPS)

S10 Fig Comparison of the p-value distributions of the original JTK_CYCLE method (with Bonferroni correction) with the empirical JTK_CYCLE method without (A) and with (B) asymmetry search. (EPS)

S11 Fig Comparison of the intersection and union of genes identified as rhythmic with Benjamini-Hochberg adjusted p-values less than 0.05 (blue bars) and 0.20 (red bars) for empirical JTK_CYCLE with different asymmetry searches. (A) The number of genes with a Benjamini-Hochberg adjusted *p*-value (FDR) below 0.05 (blue) and 0.20 (red) are shown. (B) A comparison of the intersection (below the diagonal) and union (above the diagonal) of genes identified as rhythmic with Benjamini-Hochberg adjusted *p*-values less than 0.05 for the different methods. Abbreviations are the same as in S8 Fig.
(EPS)

S12 Fig Comparison of JTK_CYCLE asymmetry search depths. Points represent genes, colored by the asymmetry search by 4 h-estimated asymmetries. The black vertical and horizontal lines mark a FDR of 0.05 (−log_10_ 0.05 ≈ 1.30). Genes to the the right of the vertical line pass the threshold cutoff for eJTK_aby4, while genes above the horizontal line pass the threshold cutoff for eJTK with asymmetry search of 8 and 16 h. Genes that are above the horizontal line but left of the vertical line barely pass the threshold and have asymmetries in the range of 8 to 16 h. Genes that are right of the vertical line but below the horizontal line pass the threshold much more significantly than the previously mentioned genes and have asymmetries that are more extreme.
(EPS)

S13 Fig Using a cosine as a reference waveform instead of a triangle does not produce substantially different results in genes identified as cycling. A comparison of the intersection and union of genes identified as rhythmic with Benjamini-Hochberg adjusted *p*-values less than 0.05 (blue bars) or 0.20 (red bars) for empirical JTK_CYCLE without asymmetry (eJTK), and empirical JTK_CYCLE with asymmetry search of 4, 8, 12, 16, and 20 h (eJTK_aby4) calculated with a reference waveform of a triangle (no prefix) or with a reference waveform of a cosine (prefix “cos”). (A) The number of genes with a Benjamini-Hochberg adjusted *p*-value below 0.05 (blue) and 0.20 (red) are shown. (B) A comparison of the intersection (below the diagonal) and union (above the diagonal) of genes identified as rhythmic with Benjamini-Hochberg adjusted *p*-values less than 0.05 for the different methods.
(EPS)

S14 Fig Using a cosine as a reference waveform instead of a triangle does not produce substantially different results in annotation terms enriched for in genes identified as cycling. Annotation terms identified as enriched by DAVID share many similarities and were therefore grouped. The number of annotation terms enriched in the genes discovered by each method are shown in grey shading and red numbers. Empirical JTK_CYCLE methods with and without asymmetry search (“_aby4” and no suffix, respectively) and with a triangle as a reference waveform or cosine as a reference waveform (no prefix or “cos”, respectively). The annotation terms displayed are enriched with Benjamini-Hochberg adjusted *p*-values below 0.05.
(EPS)

S15 Fig Comparison of genes identified as cycling by Keegan *et al*. and empirical JTK_CYCLE with asymmetry search of 4 h (eJTK_by4). (A) All the genes shown passed the ANOVA pre-screen, but only the green ones are identified as cycling by Keegan *et al*. [27]. Higher negative logarithms of *p*-values are more significant than lower ones: the horizontal black line indicates a Benjamini-Hochberg adjusted *p*-value for eJTK_aby4 of 0.05. (B) All the genes shown were identified as cycling by Keegan *et al*. The mean and variance of the genes identified as cycling by Keegan *et al*. and eJTK_aby4 (blue), are 4.34 and 0.54, respectively. The mean and variance of the genes identified as cycling by Keegan *et al*. and but not eJTK_aby4 (red), are 4.75 and 0.56, respectively. (C) All the genes shown were identified as cycling by Keegan *et al*. The asymmetry of the genes was determined by eJTK_aby4.
(EPS)

S16 Fig Comparison of genes identified as cycling by Keegan *et al*., Wijnen *et al*., and empirical JTK_CYCLE with asymmetry search by 4 h (eJTK_by4). (A) Comparison of genes identified as rhythmic by Keegan *et al*. [27], Wijnen *et al*. [32], and eJTK_by4. Stacked bars are colored to represent the asymmetry, as determined by eJTK_aby4. eJTK_aby4 identifies more genes with non-12 h asymmetries than the other methods. “Unassigned” refers to genes that were excluded from the empirical JTK_CYCLE analysis. (B) For each gene, the references on FlyBase (http://flybase.org) that mention the gene were identified. The genes identified by eJTK_by4, Keegan *et al*., and Wijnen *et al*. are shown in a histogram with stacked bars colored to represent the genes being cited by references with “circadian” in the title or abstract, genes cited in the original five dataset papers, or neither. While there are more genes uniquely identified by Wijnen *et al*., there are more total genes identified by eJTK_by4, as well as more genes that are cited in papers that have “circadian” in their title or abstract.
(EPS)

S17 Fig Z-score expression time series of *cbt*, *tws*, *Est-Q*, and *ABGE* averaged across replicate time points. (EPS)

S18 Fig KEGG pathway “dme00480: Glutathione metabolism” is enriched in genes identified as rhythmic by eJTK_aby4. Peak expression (phase) of these genes is mainly in the light period. (A) Z-scored gene expression of genes from the metadataset involved in glutathione metabolism averaged across 24 h and interpolated to every 2 h. (B) Phase and asymmetry distribution of the genes from the metadataset involved in glutathione metabolism.
(EPS)

S19 Fig Gene ontology “GO:0055114 oxidation reduction” is enriched in genes identified as rhythmic by eJTK_aby4. Peak expression (phase) of these genes is distributed over 24 h. (A) Z-scored gene expression of genes from the metadataset involved in oxidation reduction averaged across 24 h and interpolated to every 2 h. Black indicates time points where data were not available (NA). (B) Phase and asymmetry distribution of the genes from the metadataset involved in oxidation reduction.
(EPS)

S20 Fig PIR keyword “alternative splicing” is enriched in genes identified as rhythmic by eJTK_aby4. Peak expression (phase) of these genes is distributed over 24 h. (A) Z-scored gene expression of genes from the metadataset involved in alternative splicing averaged across 24 h and interpolated to every 2 h. (B) Phase and asymmetry distribution of the genes from the metadataset involved in alternative splicing.
(EPS)

S1 Data This Excel spreadsheet file contains the time series for the metadataset, with official gene names, after Z-scoring has been performed. (XLSX)

S2 Data This Excel spreadsheet file contains several pages referring to the output of the rhythm detection methods on the metadataset, as well as the DAVID results for those methods. The method results provided are all for the metadataset after pre-processing: eJTK_aby4, eJTK_a12, JTK_BF_aby4, JTK_BF_a12, cos_eJTK_aby4, cos_eJTK_a12, ANOVA, and F24.
(XLSX)

S3 Data This Excel spreadsheet file contains several pages referring to the output of the rhythm detection methods on the metadataset. The method results provided are all for the metadataset after pre-processing: JTK_BH_aby4, JTK_BH_a12 (these two come with DAVID results), eJTK_a04-12-20, and eJTK_a08-16.
(XLSX)
